# Oral squamous cell carcinoma in relation to field precancerisation: pathobiology

**DOI:** 10.1186/1475-2867-13-31

**Published:** 2013-04-03

**Authors:** Liviu L Feller, Razia RAG Khammissa, Beverly B Kramer, Johan J Lemmer

**Affiliations:** 1 Department of Periodontology and Oral Medicine, University of Limpopo, Medunsa campus, South Africa; 2 School of Anatomical Sciences, Faculty of Health Sciences, University of Witwatersrand, Johannesburg, South Africa

**Keywords:** Field precancerisation, Oral squamous cell carcinoma

## Abstract

Squamous cell carcinoma of the oral cavity evolves within a field of precancerized oral epithelium containing keratinocytes at different stages of transformation. Following acquisition of additional genetic alterations, these precancerous keratinocytes may become cancerous.

Persons with apparently successfully treated oral squamous cell carcinoma are at high risk of developing a new carcinoma at, or close to the site of the treated tumour. This second carcinoma may have developed either from malignant keratinocytes left behind at surgery (recurrence), or from transformed keratinocytes within the field of precancerized epithelium from which the primary carcinoma had arisen (new carcinoma).

The cells of the new carcinoma may have genetic changes in common with the cells of the original carcinoma because both are descended from a proliferating monoclone within the precancerized field; but if the new cancer originates from a different clone, it may have a dissimilar genetic profile even if the original and the new carcinoma are closely contiguous.

The purpose of this article is to review the pathobiology of oral squamous cell carcinoma in relation to fields of precancerised oral epithelium.

## Introduction

Squamous cell carcinoma of the oral cavity constitutes about ninety percent of all oral malignancies. It can affect any site of the oral mucosa, but most commonly the tongue and the floor of the mouth. Despite advances in diagnostic techniques and improvement in treatment modalities, the prognosis of oral squamous cell carcinoma (OSCC) remains poor, mainly owing to the high-rate of local and regional recurrence and to the development of new malignant changes within the original field of precancerisation [1-7].

Most OSCCs develop within fields of precancerized epithelium which contain keratinocytes at different stages of transformation. The persistence of such precancerized fields where there has previously been OSCC is the reason for the high-rate of occurrence of new tumours [6,8-10].

Although the literature almost invariably refers to a field of epithelium containing genetically altered keratinocytes at different stages of transformation as a field of cancerization, we prefer to term such a field, a field of precancerization. This is because only upon acquisition of additional genetic alterations, will the initially transformed precancerous keratinocytes become cancerous giving rise to a carcinoma or to multiple carcinomas. As long as a carcinoma has not become apparent in an epithelial field containing genetically altered keratinocytes, the correct term of this field is a precancerized epithelial field, and the term field of cancerization is inappropriate.

The purpose of this article is to review the pathobiological mechanisms that bring about the formation of precancerized fields of epithelium from which OSCC can arise.

### The biology of oral epithelium as it relates to OSCC

The oral epithelium is in a constant process of turnover. Thin non-keratinized epithelia such as those of the epithelium of the floor of the mouth and of the ventral surface of the tongue turn over more rapidly than do the thick keratinized epithelia, such as those of the hard palate and the gingiva. This is because the rate of proliferation of basal keratinocytes is higher in non-keratinized epithelium than in keratinized epithelium. The risk of cytogenetic mutations and subsequent cancerous transformation is greater in basal keratinocytes with a higher rate of cell division than in those with a lower rate of cell division, hence the greater risk in non-keratinized epithelium [11,12].

Proliferation of cells occurs in the basal and parabasal layers of the oral epithelium which are referred to as the progenitor cell compartment. This progenitor cell compartment comprises two functionally distinct populations of cells: a smaller population of tissue-specific stem cells, and a larger population of transient-amplifying cells [11-13]. The tissue-specific stem cells occupy a specialized niche in relation to their neighbouring cells. The stem cells contain the genomic information of the oral epithelium, are undifferentiated but have the capacity to differentiate. They divide infrequently, have the capacity for unlimited self-renewal, maintain active expression of telomerase and do not readily undergo apoptosis [11,12,14-17].

The mitotic division of a tissue-specific stem cell gives rise either to two daughter stem cells which remain in the stem cell niche or to one daughter stem cell which remains in the stem cell niche and to a second daughter transit-amplifying cell that leaves the stem cell niche, but remains in the progenitor compartment. Subsequently, the transit-amplifying cells undergo mitosis and each gives rise either to two daughter transit-amplifying cells which remain in the progenitor compartment, or to two daughter cells which begin to differentiate. Those daughter cells which exit the progenitor compartment and differentiate into keratinocytes, begin the process of maturation and gradually rise through the morphologically distinct cell layers of the epithelium, to the surface where they are shed [12,14,18].

### The origin of the OSCC precursor cell

OSCC arises by malignant transformation of a single precursor cell which by clonal expansion gives rise to a monoclonal cancer cell population. It appears that the precursor cancer cells possess the capacity for relatively unlimited self-renewal but have a limited rate of apoptosis with the outcome of longevity and the ability to initiate and sustain the ongoing growth of the cancerous tissue [18-21].

The origin of the precursor cell which gives rise to OSCC is uncertain. It is likely that it arises, as is the case in other cancers, from a tissue-specific stem cell or its progenitor cell, which has acquired epigenetic and/or genetic alterations. However, it is also possible that the OSCC precursor cell may have arisen from a stem cell which has acquired a precancerous phenotype during embryogenesis and has then differentiated into a tissue-specific cancer stem cell [14,17,22,23]. Another possibility is that the OSCC precursor cell originates from a mature keratinocyte which has undergone cytogenetic alterations resulting in its dedifferentiation into the analogue of an immature progenitor/stem cell which can express the dysregulated intracellular pathways and transcription factors of a tissue-specific cancer stem cell phenotype [14,18,24,25].

Cancer precursor cells, regardless of how they may have arisen possess the capacity for self-renewal, and hence are capable of initiating and sustaining growth of a cancer. It is likely that the OSCC comprises a heterogeneous population of cancer stem cells, cancerous transit-amplifying cells and post-mitotic cancerous cells at different stages of abnormal differentiation [14,18,24,25]. The cancer stem cells constantly provide new cancerous transit-amplifying cells which have a high proliferative rate, thus perpetuating the growth of the carcinoma. The transit-amplifying cancerous cells exhibit uncontrolled cell proliferation and prolonged survival and are the force behind tissue invasion and destruction; but owing to their limited capacity of cell renewal, they cannot alone sustain tumour growth. The post-mitotic cancerous keratinocytes at different stages of differentiation have no proliferative capacity [15,18,20,26].

Thus the overall growth of OSCC is brought about by the multiplication of cells with a cancer stem cell phenotype and by the uncontrolled proliferation of monoclonal cancerous transit-amplifying cells [18].

### Field precancerization in the mouth

**Figure 1:**
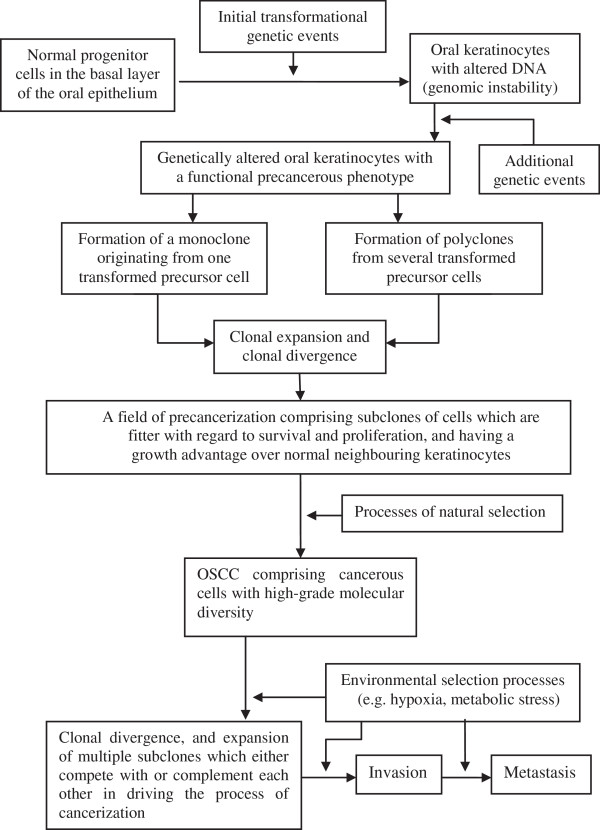
**Schematic presentation of field precancerization in relation to OSCC.** (Based on reference [30]).

It has been proposed that OSCC starts with the transformation of a limited number of normal keratinocytes. This transformation may be brought about by epigenetic and cytogenetic changes that affect cell cycle progression, DNA repair mechanisms, differentiation and apoptosis, all of which may be caused by random mutations, by exposure to any one or more of a variety of biological, chemical or physiological carcinogenic agents, or by errors in DNA repair processes. Accumulation of additional genetic events in the transformed precancerous keratinocytes may confer upon them a growth dominance over the normal neighbouring cells, resulting in their increased representation in the affected epithelium. This creates a field of precancerized epithelium (Figure 1) [8,14,17,23,27-30].

The altered keratinocytes in a field of precancerized epithelium may be genetically similar if they have arisen from a single transformed progenitor cell which underwent clonal expansion and subsequent clonal divergence, or may be genetically dissimilar if they have arisen from different transformed progenitor cells. This would result in the evolution of subclones of keratinocytes which have had multiple episodes of cytogenetic and epigenetic alterations. In both cases the transformed clones spread laterally at the expense of the surrounding normal keratinocytes creating a field of precancerization. The size of the field increases slowly at a rate determined by the rate of cellular multiplication in that particular micro-environment, and may reach several centimetres in diameter [6,8,14,23].

Thus, a large number of genetically altered keratinocytes of similar or dissimilar clonal origin make up a field of precancerized epithelium which clinically appears normal but microscopically may show epithelial dysplasia, or in time may give rise to precancerous lesions (leukoplakia, erythroplakia) and can be discrete from, or contiguous with other precancerized fields in the affected anatomical site (Figure 1) [8,14].

The genomic instability which brings about the initial transformational events may confer upon the transformed keratinocytes in the precancerized epithelial field a phenotype which predisposes them to a heightened risk of additional genetic mutations. Ultimately, this mutator phenotype may drive the affected cells towards acquisition of a complete set of genetic alterations of a cancerous phenotype, characterized by progressive uncontrolled cellular growth [8,9,14,23,31-36].

However, it has also been proposed that the initiating events in the transformation of a normal keratinocyte to a cancerous keratinocyte, as well as the genetic events sustaining tumour growth, may be brought about by local evolutionary natural selection rather than by the mutator phenotype, thus promoting cytogenetic instability [37-39]. According to this concept, carcinogenesis usually starts with a normal rate of mutation. Subsequent repetitive rounds of mutations together with selection and clonal expansion confer upon the transformed cells a selective growth advantage. This process does not necessitate an increase in mutation rate (a mutator phenotype) [37]. Indeed, in early transformational events associated with the formation of a precancerized field, factors such as population density of transformed cells rather than the mutator phenotype of the cells may play an important role [40]. Subsequently, environmental selection pressures act on the genetically heterogeneous clonal cell population of the precancerized field driving the evolution of subclones of cells which are fitter with regard to survival and proliferation, and consequently have a growth advantage over normal neighbouring cells [39,41].

### Field precancerization in relation to OSCC

The number and combination of genetic alterations which are necessary and sufficient to initiate OSCC, and the sequence in which such alterations occur, are unknown. However, it is probable that evolutionary processes will favour mutation of genes which allow dysregulation of the cell cycle checkpoints, thus promoting increased cell proliferation. This will confer upon the transformed cells a growth advantage [41].

The probability of progression of precancerous progenitor keratinocytes to cancer cells varies in different precancerized fields, and it is impossible with confidence to distinguish histomorphologically or genetically between those keratinocytes that are destined to remain stable and those that are destined to acquire a full cancerous phenotype [14].

Furthermore, although carcinomatous transformation of normal progenitor keratinocytes is brought about by well recognised cytogenetic alterations, currently there are no reliable molecular or genetic predictors of carcinomatous transformation of precancerized keratinocytes [14,42-45]. At present, high-grade epithelial dysplasia, DNA aneuploidy and loss of heterozygosity at certain defined chromosomal loci, are associated with a greater risk of carcinomatous transformation [14,45-51].

In those fields of oral epithelium in which the precancerized keratinocytes are already committed to the pathway of cancerization, progression to cancer may vary from 6 months to 8 years. However, the factors which determine the speed of the transformation are unknown [14,46,48].

Once a carcinoma has evolved, the molecular diversity of cancerous cells within a single tumour [52] drives the evolution of subclones of cancerous cells via the process of natural selection [53]. The cell population of each of the subclones within the tumour microenvironment harbours distinct genetic profiles with specific biological properties [53,54]. In response to environmental selection pressures such as hypoxia or metabolic stresses, the intratumour genetic heterogeneity favours the evolution and survival of subclones of cells which will than have a growth advantage [55]. The multiple subclones occupy distinct regional niches within the tumour microenvironment [52] and may either compete with each other in which case the dominant subclone will drive the process of carcinogenesis or they may complement each other in which case the subclones together will drive the process of carcinogenesis (Figure 1) [53]. Common genetic alterations found in all cancerous cells of the subclones are probably the outcome of initial genetic events while less common genetic alterations represent secondary genetic events [52].

The existence of an epithelial field of precancerization may explain several aspects of the pathobiological behaviour of OSCC. Firstly, it can explain why many cases of OSCC arise from, or are closely contiguous with precancerous lesions such as oral leukoplakia or erythroplakia; secondly, it can explain why apparently normal-looking epithelium surrounding OSCC harbours cells with genetic alterations and/or cells with dysplasia. This may account for the high-rate of recurrence of OSCC despite apparently successful excision. Lastly, the existence of an epithelial field of precancerisation may also explain why more than one OSCC not uncommonly may manifest synchronously or metachronously, and may have similar or dissimilar molecular profiles depending upon whether they developed from subclones descended from a single monoclone or from polyclones [14].

### Genomic instability as it relates to fields of precancerization

The cellular genomic integrity of keratinocytes is maintained by various mechanisms including DNA monitoring and repair, checkpoints that regulate the cell cycle and genes that ensure the accurate replication and segregation of chromosomes during mitosis. Malfunction of these mechanisms may either bring about genomic instability which is associated with an increased risk of acquiring additional genetic alterations; or it may confer upon the transformed keratinocytes growth advantage over the normal neighbouring keratinocytes that can ultimately culminate in carcinoma [20,28,29,56-58].

### Defects in DNA repair mechanisms

Damage to DNA in keratinocytes may be caused by many factors including spontaneous mutations, errors in DNA replication brought about by misincorporation of nucleotides by DNA polymerases, or by a variety of exogenous and endogenous mutagens [57,58]. If the damage to DNA affects critical genes and exceeds the cell’s repair capacity, the altered DNA may propagate by cell division, promoting cell transformation and carcinogenesis.

Normally, DNA nucleotide-excision repair (NER) corrects covalent alterations to DNA induced by chemical mutagens [58], and by ultraviolet-induced helix–distorting lesions of DNA [59]. Functional defects in NER genes in keratinocytes bring about an accumulation of mutations that are associated with an increased risk of SCC, particularly of the skin [59].

Genetic or epigenetic inactivation of the DNA mismatch repair mechanisms which normally function in correcting DNA replication errors may result in the formation of changes in the lengths of the repetitive nucleotide sequences. This occurs mainly in DNA segments between genes (microsatellite sequences) and is a specific type of genomic instability termed microsatellite instability [57]. Microsatellite instability may also be brought about by inherently error-prone DNA polymerases which during DNA replication generate errors of a magnitude that exceeds the normal capacity for correction by mismatch repair [60]. In turn, the mutated microsatellite sequences may cause inactivation of tumour suppressor genes [61]. Thus, as mutated repetitive sequences can be found within coding regions of growth regulatory genes, microsatellite instability can promote mutagenesis [61].

While in hereditary cancers, mutations in DNA repair genes are common, in sporadic non-hereditary cancers mutations in DNA repair genes are infrequent, and if present, probably represent late carcinogenic events [62]. For example, molecular alterations in Fanconi anemia/BRCA intracellular signalling pathways that regulate cellular repair of DNA damage are a risk for OSCC [63]. OSCC in association with Fanconi anemia occurs in a significantly higher frequency and at a younger age than the occurrence of oral squamous cell carcinoma in the general population. Functional inactivation of Fanconi anemia/BRCA pathways are also observed in some cases of sporadic OSCC [63-65].

### Oncogenes and tumour suppressor genes (anti-oncogenes)

In sporadic cancers, the genomic instability is characterized by molecular alterations of oncogenes, of tumour suppressor genes (anti-oncogenes), and/or of DNA checkpoint control genes. Increased activity of oncogenes results in excessive cell proliferation with consequent increase in DNA damage. Tumour suppressor genes function as anti-oncogenes regulating intracellular growth signal pathways. DNA checkpoint control genes ultimately induce cell death in response to DNA damage which exceeds the repair capacity of the cells. This prevents propagation of altered DNA to daughter cells. Some tumour-suppressor genes (e.g. p53) are also involved in arrest of the cell cycle and in apoptosis [62,66].

Mutations in oncogenes conferring cellular self-sufficiency in growth signals, or in tumour suppressor genes resulting in dysfunctional anti-oncogenic activity, or mutations in both, may induce deviations in DNA replication, leading to accumulation of DNA damage and consequent genetic instability. This process is not sufficient to induce a complete set of genomic alterations of a cancerous phenotype, but can be an initiating event in carcinogenesis, establishing a state of precancerization [62,67]. Overexpression of numerous oncogenes including epidermal growth factor and its ligand, transforming growth factor-α, and loss of function of several tumour-suppressor genes including TP53, TP16 and NOTCH1 have been implicated in the pathogenesis of OSCC [66,68-70]. It appears that carcinomatous cells of squamous cell carcinomas of the head and neck contain more inactivating mutations in tumour suppressor genes (anti-oncogenes)than activating mutations in oncogenes [70].

Normally, excessive DNA damage will activate DNA checkpoint control genes (e.g. p53), mediating apoptosis. However, later in the chain of genetic events of carcinogenesis, loss of function of DNA checkpoint control genes owing to evolutionary selection pressures or to carcinogen-induced mutations, or to sporadic mutations, will enable the initially transformed cells to evade apoptosis, and ultimately to acquire a cancerous phenotype [62].

Human papillomavirus (HPV)-negative squamous cell carcinoma of the head and neck (HNSCC) is burdened with more genetic alterations than HPV-positive HNSCC; and HNSCC-associated with tobacco, is burdened with more mutations than HNSCC not related to tobacco use [70].

### Chromosomal instability

Chromosomes of cancer cells are unstable [62,71], characterised by diverse chromosomal aberrations including aneuploidy, deletions, translocations, amplifications and are subject to abnormal mitoses [56-58,60-62]. Chromosomal instability may be brought about by non-specific chromosomal errors (aneuploidy), by specific mutations of mitotic control genes, or by evolutionary selection pressures imposed by the somatic environment. In turn, the chromosomal errors impose selection pressures that favour molecular alterations in mitotic checkpoint genes, allowing the replication of cells with chromosomal alterations. Had there not been loss of function of mitotic checkpoint control genes, the cells harbouring chromosomal errors would have undergone apoptosis [71].

Large-scale genomic gains or losses may result in over-expression of oncogenes or in loss of function of tumour suppressor genes, thus promoting carcinogenesis [72]. The loss of heterozygosity which is a loss of paternal or maternal alleles within chromosomal regions with tumour suppressor genes occurs frequently in transformed keratinocytes of potentially malignant oral lesions. Loss of heterozygosity at specific chromosomal regions in these keratinocytes (e.g. 3p, 9p, 17p, 13q, 18q) is associated with progression of potentially malignant lesions to oral SCC [14,46,47,50,68,72,73].

Aneuploidy involving chromosomes that have genes governing mitosis destabilizes the karyotype, causing a degree of chromosomal instability which is proportional to the degree of aneuploidy [74]. The risk of potentially malignant oral lesions progressing to OSCC is thus much greater for those lesions manifesting aneuploidy than for those with diploidy [14,51,75,76]. Indeed, it has been reported that about 80% of OSCCs harbour DNA aneuploidy [77], and that acquisition of aneuploidy is an early genetic event in oral carcinogenesis [78].

It is not clear whether chromosomal instability is the driving force of carcinogenesis or merely a by-product, and whether it occurs as a consequence of the high rate of mutations in specific genes maintaining the stability of cellular genetic material; or whether these mutations only evolve secondarily to the chromosomal instability [71,79]. Nevertheless, it is accepted that the acquisition of chromosomal instability is an early event in carcinogenesis [80,81].

## Summary

Most OSCCs develop in fields of precancerized epithelium in which there is clonal expansion of phenotypically normal but genetically altered keratinocytes. These genetically unstable precancerous keratinocytes manifest aneuploidy, gain or loss of chromosomal material, or alterations in the sequences of nucleotides. The genomic instability favour further acquisition of genetic alterations leading to growth superiority or inferiority of the affected cells. The genetically advantaged cells may ultimately acquire a cancerous phenotype.

## Competing interests

The authors declare that they have no competing interests.

## Authors’ contributions

The concept of the paper was devised by LF. LF, RAGK, BK and JL wrote the manuscript. All authors read and approved the final manuscript.
